# The association between sex and neonatal respiratory distress syndrome

**DOI:** 10.1186/s12887-024-04596-3

**Published:** 2024-02-19

**Authors:** Keren Fang, Shaojie Yue, Shuo Wang, Mingjie Wang, Xiaohe Yu, Ying Ding, Mei Lv, Yang Liu, Chuanding Cao, Zhengchang Liao

**Affiliations:** Department of Neonatology, Xiangya Hospital, Central South University, Xiangya Road No.87, Changsha, Hunan Province 410008 China

**Keywords:** Sex, Respiratory distress syndrome, Neonate, Risk, Logistic regression

## Abstract

**Background:**

To investigate the association between sex and neonatal respiratory distress syndrome (NRDS).

**Methods:**

Neonates born at our hospital and transferred to the neonatal department within 1 h were retrospectively analyzed. Depending on whether they developed NRDS during their hospital stay, the neonates was divided into NRDS and non-NRDS groups. There were 142 neonates in the NRDS group (95 males and 47 females) and 310 neonates in the non-NRDS group (180 males and 140 females). The neonates’ data on gestational age (GA), sex, birth weight, white blood cell count (WBC), platelet count (PLT), C-reactive protein (CRP), total immunoglobulin M (total IgM), gestational diabetes mellitus(GDM), antenatal steroids use, meconium-stained amniotic fluid, and preterm premature rupture of membranes(PPROM) were gathered.

**Results:**

452 neonates (265 males and 187 females) were involved for the purpose of collecting basic characteristic. Multivariate analysis, males had a 1.87 times higher risk of NRDS than females (*P* < 0.05) after controlling for the confounding effects of GA, birth weight, WBC, PLT, CRP, total IgM, GDM, antenatal steroids use, meconium-stained amniotic fluid, and PPROM.

**Conclusions:**

Sex was associated with NRDS; males had a considerably higher risk of NRDS than females.

## Background

Neonatal respiratory distress syndrome (NRDS), generally known to as neonatal pulmonary hyaline membrane disease, is the term used to describe progressive dyspnea and respiratory failure in neonates shortly after birth. Clinical signs of NRDS include shortness of breath, irregular breathing patterns, and apnea. This condition is often caused by gradual alveolar atrophy brought on by a shortage of pulmonary surfactant. Lack of early detection may cause the higher risk of NRDS, which could result in complications such chronic lung disease, respiratory failure, and even death [1, 2]. According to research on the causes of neonates' death in China, 42.86% of them died from NRDS [3]. It has been reported that sex may have an impact on the progresses of diseases. Han et al. [4] reported in their research on the relationship between pulmonary diseases and females that females had higher rates of hospitalization and mortality than males in diseases such as asthma, pulmonary hypertension and lymphatic smooth muscle tumors; DeMeo et al. [5] found that in a retrospective analysis of a large sample of multicenter chronic obstructive pulmonary disease (COPD) patients in the United States, young females with COPD were more likely to have more severe dyspnea, airflow limitation, and a greater risk of exacerbation than males with COPD; Lindsay A. et al. [6] observed that males had a greater risk of lymphocytic leukemia, Hodgkin’s lymphoma, Burkitt’s lymphoma, and other non-Hodgkin lymphoma in research of children in the United States. Whether there is an independent effect of sex in NRDS is not currently reported to describe and quantify the risk of NDRS by sex. This research aims to find out whether there is a possible linear link between sex and NRDS, to offer a reference for comprehending sex differences in NRDS and their clinical significance, and to assist pediatrician in formulating appropriate diagnostic procedures and treatment strategies according to the sex of the patients.

## Methods

### Subjects

By retrospectively analyzing 820 neonates born in our hospital and admitted to the neonatal unit (NICU) within 1 h from January 2020 to December 2020, indicators such as gestational age (GA), sex, birth weight, white blood cell count (WBC), platelet count (PLT), C-reactive protein (CRP), total immunoglobulin M (IgM) in neonatal serum, gestational diabetes mellitus(GDM), antenatal steroids use, meconium-stained amniotic fluid, and preterm premature rupture of membranes(PPROM) were collected, and 452 neonates were finally included. The groups were based on whether they progressed to NRDS during hospitalization: there were 142 neonates in the NRDS group, including 95 males and 47 females with an average age of 0.40 (0.30–0.50) hours; there were 310 neonates in the non-NRDS group, including 170 males and 140 females with an average age of 0.50 (0.50–0.67) hours.

### Measurements

#### Inclusion and exclusion criteria

**Fig. 1:**
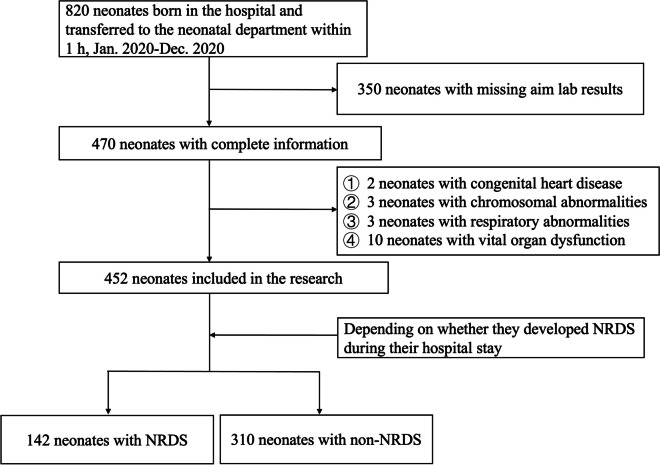
Flow chat

Neonates who were born in the hospital and transferred to the neonatal department within 1 h were included. Neonates who had other illnesses like congenital heart disease or chromosomal abnormalities, had respiratory abnormalities, with vital organ dysfunction or lacked the aim lab results were excluded (Fig. 1).

### Data analysis

For measurement data, mean ± SD/Median (Q1, Q3) was used, and for count data, n (%) was used, and t-test/χ2 test/Kruskal Wallis test was used for comparison between groups. Univariate and multifactorial Logistic regression were used to analyze the possible linear relationship between sex and NRDS, and three models were constructed to illustrate the stability of this relationship: model 1 adjusted for none; model 2 adjusted for demographic indicators; and model 3 was a fully adjusted model. Statistical analyses were performed with the R package (version 3.6.1) (http://www.R-project.org, The R Foundation) and EmpowerStats (http://www.empowerstats.com, X&Y Solutions, Inc, Boston, MA) were performed. *P* < 0.05 (two-sided) was considered a statistically significant difference.

### Ethics statement

The studies involving human participants were reviewed and approved by the Ethics Committee of our hospital, our university [202112258]. Written informed consent to participate in this study was provided by the participants’ legal guardian/next of kin.

## Results

### Comparison of general characteristics

A total of 452 neonates, 265 males and 187 females, with an average age of 0.50 (0.42–0.50) hours, were included in this research. GA, Sex, birth weight, WBC, PLT, antenatal steroids, meconium-stained amniotic fluid, and PPROM were different between the two groups and the differences were statistically significant (*P* < 0.05) (Table 1).

**Table 1:** Basic characteristic of neonates born in the non-NRDS group compared to the NRDS group [*n *= 452, Mean SD/ Median (Q1, Q3)/ n (%)]

Groups	GA（weeks）	Sex		Birth weight （100g)	CRP（mg/L)	Total IgM（mg/L)	WBC (*10^9/L)	PLT (*10^9/L)	GDM		Antenatal steroids		PPROM			Meconium-stained amniotic fluid	
		Female	Male						No	Yes	No	Yes	No	<18 hours	>18 hours	No	Yes
Non-NRDS group (*n *= 310)	37.04 ± 2.68	140 (45.16)	170 (54.84)	27.76 ± 7.36	3.12 (1.85-7.77)	86.60 (66.10-115.00)	14.25 (11.28-18.10)	247.37 ± 65.45	286 (92.26%)	24 (7.74%)	47 (15.16%)	263 (84.84%)	245 (79.03%)	29 (9.35%)	36 (11.61%)	68 (21.94%)	242 (78.06%)
NRDS group (*n *= 142)	31.56 ± 2.71	47 (33.10)	95 (66.90)	16.57 ± 5.58	3.16 (1.97-5.99)	76.90 (52.40-107.00)	10.30 (6.93-14.33)	221.11 ± 72.66	128 (90.14%)	14 (9.86%)	77 (54.23%)	65 (45.77%)	88 (61.97%)	9 (6.34%)	45 (31.69%)	8 (5.63%)	134 (94.37%)
*P*-value	<0.001	0.016		<0.001	0.298	0.431	<0.001	<0.001	<0.001		<0.001		<0.001			<0.001	

*CRP* C-reactive protein, *Total IgM* total immunoglobulin M, *WBC* white blood cell count, *PLT* platelet count, *GDM* gestational diabetes mellitus, *PPROM* preterm premature rupture of membranes

### Univariate analysis

GA, birth weight, WBC and PLT were negatively associated with NRDS, the risk of NRDS decreased by 51%, 22%, 6% and 1% for each unit increase in GA, birth weight, WBC and PLT, respectively (All *P* < 0.05); Antenatal steroids were negatively associated with NRDS, with a 75% reduction in the risk of NRDS with the use of antenatal steroids (*P* < 0.05). Sex was positively associated with NRDS, the risk of NRDS was 67% higher in males than in females (*P* = 0.016). PPROM > 18 h and meconium-stained amniotic fluid were also associated with an increased risk of NRDS by 2.48 times and 3.71 times, respectively (All *P* < 0.05). CRP/ total IgM/GDM showed a positive trend of association with NRDS and may be a potential risk factor, but the difference was not statistically significant (Table 2).

**Table 2:** Univariate analysis of each factor with NRDS [*n* = 452, Mean ± SD/ Median (Q1, Q3)/ n (%)]

	Statistics	OR (95%CI)	*P*-value
GA (Weeks)	35.32 ± 3.70	0.49 (0.43, 0.56)	0.000
Sex			
Females	187 (41.37%)	1.0	
Male	265 (58.63%)	1.67 (1.10, 2.52)	0.016
Birth weight (100 g)	24.25 ± 8.60	0.78 (0.74, 0.82)	0.000
CRP (mg/L)	3.13 (1.88–7.45)	1.01 (0.99, 1.02)	0.305
Total IgM (mg/L)	83.00 (61.38–114.75)	1.01 (1.00, 1.01)	0.436
WBC (*10^9/L)	13.25 (9.88–16.95)	0.94 (0.90, 0.97)	0.000
PLT (*10^9/L)	239.09 ± 68.82	0.99 (0.99, 1.00)	0.000
GDM			
No	414 (91.59%)	1.0	
Yes	38 (8.41%)	1.30 (0.65, 2.60)	0.453
Antenatal steroids			
No	124 (27.43%)	1.0	
Yes	328 (72.57%)	0.15 (0.10, 0.24)	0.000
PPROM			
No	333 (73.67%)	1.0	
< 18 h	38 (8.41%)	0.86 (0.39, 1.90)	0.716
> 18 h	81 (17.92%)	3.48 (2.11, 5.75)	0.000
Meconium-stained amniotic fluid			
No	76 (16.81%)	1.0	
Yes	376 (83.19%)	4.71 (2.20, 10.09)	0.000

Result variables: NRDS or non-NRDS

Exposure variables: gestational age, sex, birth weight, CRP, total IgM, WBC, PLT, GDM, antenatal steroids, PPROM, meconium-stained amniotic fluid

Adjusted: None

*CRP* C-reactive protein, *Total IgM* total immunoglobulin M, *WBC* white blood cell count, *PLT* platelet count, *GDM* gestational diabetes mellitus, *PPROM* preterm premature rupture of membranes

### Multifactorial logistic regression

Compared to model 1, males had a higher risk of NRDS than females in model 2, but the connection was remained statistically significant. Model 3 showed a 1.87 times greater risk of NRDS in males compared to females, which was independent after correcting for GA, birth weight, WBC, PLT, CRP, total IgM, GDM, antenatal steroids, PPROM and meconium-stained amniotic fluid (*P* < 0.05). This suggests a strong and consistent effect of sex on NRDS and less influence from factors other than sociodemographic characteristics (Table 3).

**Table 3:** Comparison of different models of sex and NRDS

	OR (95%CI)	
	Female	Male
Model 1	1.0	1.67 (1.10, 2.52)
*P*-value		0.016
Model 2	1.0	2.79 (1.47, 5.33)
*P*-value		0.002
Model 3	1.0	2.87 (1.42, 5.80)
*P*-value		0.003

Result variables: NRDS or non-NRDS

Exposure variable: sex

Model 1 adjusted: none

Model 2 adjusted: gestational age, birth weight

Model 3 adjusted: gestational age, birth weight, CRP, total IgM, WBC, PLT, GDM, antenatal steroids, PPROM, meconium-stained amniotic fluid

*CRP* C-reactive protein, *Total IgM* total immunoglobulin M, *WBC* white blood cell count, *PLT* platelet count, *GDM* gestational diabetes mellitus, *PPROM* preterm premature rupture of membranes

## Discussion

NRDS occurs in progressive exacerbation immediately after birth, primarily in preterm neonates, with a higher frequency at lower GA and more severe complications, and has been the subject of widespread concern in families and hospitals. The prerequisites for treating patients with NRDS are to avoid invasive tracheal intubation wherever possible, to diagnose and intervene early in neonates with NRDS, and to administer pulmonary surfactant as soon as possible, which may have a prognostic benefit, maximize survival, and minimize potential adverse effects [7].

Sex was discovered to be a factor in this research’s findings, with males substantially more at risk for NRDS than females. In retrospective analytic research of a sizable sample of italian neonatal hospitalization data, Condò et al. [8] revealed that birth weight and sex also presented a risk effect on NRDS. The male disadvantage in NRDS was reported by Laube et al. [9] in research of antenatal glucocorticoids stimulation of alveolar cell epithelial Na^+^ transport. However, antenatal glucocorticoids administration had no sex-specific effects on Na^+^ transport, indicating that antenatal glucocorticoids administration had no effect on the male disadvantage in NRDS. Wen et al. [10] conducted retrospective research in Taiwan with a sample size of 13490 people and discovered that males were more likely than females to experience NRDS under the assumption that the mother had pre-eclampsia. WBC, CRP and total IgM are all infection-related indicators, of which total IgM indicates a recent infection and IgM does not pass through the placenta, when IgM is increased, it suggests that the fetus may be infected [11]. There were no large fluctuations in risk between model 2 and model 3 in this research, suggesting that the risk of NRDS was not increased by adjusting for either potential infection-related factors (WBC/CRP/ total IgM) or maternal status, and was more influenced by demographic factors (GA/weight).

The mechanism via which sex influences the risk of NRDS is unknown, and there are two probable explanations: (1) Male fetuses are significantly more likely than female fetuses to be born prematurely, and their average GA is significantly lower than that of their female counterparts. Additionally, there is a negative correlation between the incidence of NRDS and gestational age [12]; and (2) there may be a connection between sex hormone action during lung development [13]. Androgens include dehydroepiandrosterone, androstenedione and testosterone, and the main androgen in male is testosterone, which is produced by testicular mesenchymal cells and released into the circulatory system. In selected target tissues, testosterone is reduced to 5α-dihydrotestosterone, which is considered to be the most potent natural androgen [14], and testosterone can be secreted by embryonic-type mesenchymal stromal cells during fetal life, consistent with Lee et al. [15] who found that androgens can increase the activity of the EGF pathway by activating the SRC to delay fetal alveolar type II (AT II) epithelial cells maturation. It is generally believed that inadequate pulmonary surfactant and immaturity of AT II cells are what cause the development of NRDS [16]. It is therefore reasonable to assume that testosterone, which can be secreted during fetal life, may cause immaturity of AT II cells and consequently increase the risk of NRDS in male neonates. According to Kim et al. [17] research’s, androgens’ inhibitory action on AT II cells is the primary cause of male sexual increased risk of NRDS. The risk of NRDS will be reduced as a result of the relatively high estrogen levels in females, which will encourage the production of pulmonary surfactant and increase the number of AT II cells. Stylianou-Riga et al. [18] found that in retrospective research of 134 neonates born by cesarean section, the incidence of NRDS in male neonates was almost 3 times higher than that of female neonates. The protective impact of females may be explained by the influence of estrogen on enhancing alveolar development and pulmonary surfactant synthesis, while prolonged testosterone exposure in utero inhibits pulmonary surfactant formation in male embryos, which may explain the protective. Furthermore, Savchuk I et al. [19] discovered that during 6–7 weeks of gestation, testosterone can be detected in embryos and quickly reaches a maximum at 11–14 weeks. Human lung development started at 4 weeks of gestation and proceeds through the pseudoglandular stage, which last from 7 to 17 weeks of gestation. During this stage, cuboidal cells formed in the distal region and were filled with glycogen, which was an essential component of pulmonary surfactant the distal cuboidal cells represented the immature AT II cells [20]. It is likely that peak testosterone affects the synthesis of glycogen in immature AT II cells during the pseudoglandular stage, which in turn affects the production of pulmonary surfactant.

The course of several clinical disorders has drawn attention to sex disparities. According to Tramunt et al. [21], new diabetes treatment protocols should be examined for the metabolic features of various sexes, and the impact of patient sex on therapy should be taken into account in the individualized management of diabetes; Rafikov et al. [22] discovered that female patients are more likely to have pulmonary arterial hypertension (PAH), perhaps due to The downregulation of BMPR2 signaling is closely associated to the development of PAH, while the male Y chromosome-specific transcription factor sex-determining region Y positively regulates the BMPR2 promoter, which upregulates BMPR2, so males may have a protective impact, suggesting that sex should be considered in the prevention and treatment of PAH. Gai et al. [23] noted that bacterial lipopolysaccharide (LPS) has a protective impact on asthma, and serum LPS levels were lower in female asthmatics than in male asthmatics, suggesting that LPS may be an important cause of phenotypic differences in asthmatics by sex, and this result may present a target for precise treatment of female asthmatics; Zhao et al. [24] observed that congenital heart disease is more common in female neonates, which may suggest sonographer to perform more careful screening when completing cardiac ultrasound in female neonates to detect the disease as early as possible.

There are also shortcomings in this research, such as single center and small sample size, and lack of evidence on the specific reasons for the differences by sex, which will be improved in future studies.

## Conclusion

The development of early NRDS may be sneaky, sex may be indicative of the onset of early NRDS, and knowledge of sex differences in NRDS may aid in the creation of effective diagnostic procedures and treatment plans tailored to the patient’s sex.

## Data Availability

The datasets generated during and/or analyzed during the current study are not publicly available due to patient privacy protection but are available from the corresponding author on reasonable request.

## Abbreviations

NRDS: Neonatal respiratory distress syndrome
CRP: C-reactive protein
IgM: Immunoglobulin M
WBC: White blood cell count
PLT: Platelet count
GDM: Gestational diabetes mellitus
PPROM: Preterm premature rupture of membranes
